# Conduction disorders after transcatheter aortic valve implantation: A comparison between SAPIEN 3 and SAPIEN 3 Ultra balloon-expandable valves

**DOI:** 10.3389/fcvm.2022.922696

**Published:** 2022-11-02

**Authors:** Giovanni Monizzi, Paolo Olivares, Giulio Makmur, Franco Fabbiocchi, Luca Grancini, Angelo Mastrangelo, Cristina Ferrari, Stefano Galli, Piero Montorsi, Antonio L. Bartorelli

**Affiliations:** ^1^Centro Cardiologico Monzino, IRCCS, Milan, Italy; ^2^Department of Clinical Sciences and Community Health, University of Milan, Milan, Italy; ^3^Department of Biomedical and Clinical Sciences, University of Milan, Milan, Italy

**Keywords:** conduction disorders in new-generation balloon-expandable valves TAVI, conduction disorders, aortic stenosis, TAVI, pacemaker

## Abstract

**Background:**

Conduction disorders (CD) are the most common complications after Transcatheter Aortic Valve Implantation (TAVI). The last generation of Edwards balloon expandable valves, the SAPIEN 3 Ultra (S3U), is provided with an external sealing skirt that aims to further reduce paravalvular leakage (PVL) compared to SAPIEN 3 (S3) and could potentially lead to higher CD rate. We sought to investigate the rate of new-onset CD in patients undergoing TAVI with the S3 or S3U valve.

**Methods:**

We included 582 consecutive patients undergoing TAVI in a single high-volume Center. Patients with previously implanted pacemaker and Valve in valve procedures were excluded. CD rate was evaluated early after implantation and at discharge.

**Results:**

No significant difference in the overall CD rate was found between S3 and S3U patients both immediately after the procedure (S3 45.5% vs. S3U 41.8%, *p* = 0.575) and at discharge (S3 30.4% vs. S3U 35.6%, *p* = 0.348) with low rate of permanent pacemaker implantation (S3 6.3% vs. S3U 5.5%, *p* = 0.749). No significant differences were found also in patients with pre-existing atrial fibrillation (S3 8.2% vs. S3U 5%, *p* = 0.648). A significantly lower rate of PVL was found with S3U compared to S3 (S3 42% vs. S3U 26%, *p* = 0.007). According to the manufacturer’s guidelines we confirmed that S3U were implanted in a significantly higher position compared to S3 (S3 4.89 ± 1.57 mm vs. S3U 4.47 ± 1.36 mm, *p* = 0.001).

**Conclusion:**

No significant difference in the rate of CD, including the need for PPM implantation, was found in patients undergoing TAVI with the S3 compared to S3U. Moreover, S3U significantly reduced the PVL rate.

## Introduction

Transcatheter aortic valve implantation (TAVI) is nowadays a worldwide accepted option for treating patients with severe aortic valve stenosis in patients at all levels of surgical risk (1–3). Conduction disorders (CD) are one of the most common complications of TAVI. Indeed, about one third of patients present CD at discharge, with the left bundle branch block (LBBB) being the most frequent (4–6). The SAPIEN 3 Ultra transcatheter heart valve (THV) (Edwards Lifesciences, Irvine, CA) is the latest iteration of the balloon-expandable Edwards THV family featuring an improved external sealing skirt that aims to further reduce paravalvular leakage (PVL) (7). A recent retrospective study comparing the Edwards SAPIEN 3 (S3) to SAPIEN 3 Ultra (S3U) valves did not find any difference in terms of 30-day clinical outcomes except for a lower rate of major vascular complications (11.4% vs. 4.5%, *p* = 0.05) and PVL with the S3U (8). Comparative studies specifically focusing on the evaluation of all types of CD after S3 or S3U valve implantation are not currently available. Thus, the primary endpoint of our study was to compare the rate of new-onset CD in patients undergoing TAVI with the S3 or S3U valve.

## Materials and methods

### Population

We prospectively included 582 consecutive patients with severe aortic valve stenosis undergoing TAVI after local Heart Team decision with a balloon-expandable S3 or S3U in a single high-volume TAVI Center (Centro Cardiologico Monzino, Milan, Italy) between January 2016 and November 2020. S3 valves were implanted from January 2016 until April 2019, and S3U valves from March 2019 onward. Experienced operators performed TAVI according to the local protocol. All subjects gave written informed consent. Exclusion criteria of the study were:

1. “Valve-in-valve” procedures (THV implantation into a previously surgical or percutaneous implanted aortic prosthesis);
2. Presence of a previously implanted pacemaker;
3. Electrocardiogram not available or not analyzable before TAVI.

**FIGURE 1:**
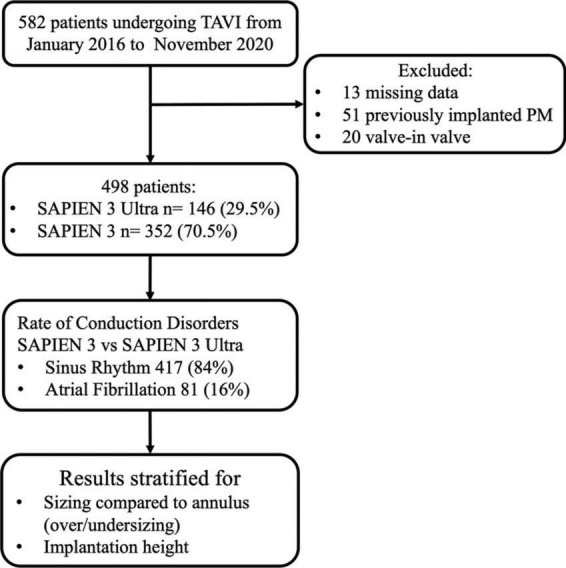
Study flowchart.

The final population of our study consisted of 498 patients. Among them, 352 (70.5%) received a S3 and 146 (29.5%) a S3U (Figure 1).

### Multislice CT scan evaluation

As recommended by the current guidelines, an ECG-gated multislice CT study (MSCT) was performed to obtain information about anatomical predictors of CD as previously demonstrated (9). For this reason aortic annulus dimension, degree of leaflet calcification, Left ventricle outflow tract (LVOT) calcifications, Membranous septum length (MSL) in addition to anatomy of the access site and peripheral vessels were collected (3, 9). A dedicated protocol was formulated, with 100–120 kV and tube current modified according to the patient’s size.

The following variables were analyzed for each patient:

1. Aortic valve calcification was quantified with MSCT according to current criteria (grade 1–4 calcification of the aortic cusps) (10).
2. The MSL was measured by two expert CT operators (PO, AM) determining the thinnest part of the interventricular septum on axial images as previously validated (11).
3. The THV implantation depth within the left ventricular outflow tract was evaluated by angiographic standard projections during the implantation. The distance between the inferior edge of the cobalt-chromium THV frame and the left and non-coronary cusps was assessed and the mean value of the two measurements was recorded (11, 12) (Supplementary Figure 4).
4. Prosthesis sizing was calculated as the ratio of THV nominal area and aortic annulus area measured with MSCT. Valve undersizing was defined as a prosthesis nominal area 5% smaller than the annular area measured with MSCT, while oversizing was defined as a nominal area 5% larger than the annular area measured with MSCT as previously validated (13). Values comprised in this interval were defined as matched THV sizing.

### Procedural evaluation

Most of the patients were treated under general anesthesia, while in some selected cases deep sedation was used as deemed indicated by the Heart Team. All baseline, procedural, and post-operative data were retrospectively recorded. Post-TAVI transthoracic echocardiography (TTE) were performed by experienced echocardiographers who are independent from TAVI operators. PVL was graded as mild, moderate, and severe according to the Valve Academic Research Consortium 3 (VARC-3) criteria.

Periprocedural complications were defined according to the Valve Academic Research Consortium-3 criteria (VARC-3) (14).

### Electrocardiographic analysis

Standard 12-lead ECG was recorded at a speed of 25 mm/s and a calibration of 1 mV/mm at baseline (within 24 h prior to the procedure), immediately after the procedure, and daily until hospital discharge. All ECGs were digitalized and reviewed by two expert cardiologists (PO, GM) blinded to the clinical data. The diagnosis of AV and intraventricular CD was based on the recommendations of the American Heart Association/American College of Cardiology Foundation/Heart Rhythm Society (AHA/ACCF/HRS) for the standardization and interpretation of ECG (15, 16).

Patients with atrial fibrillation (AF) were excluded from the evaluation of new-onset AV block and included in the assessment of permanent pacemaker (PPM) implantation rate.

The variables analyzed in each ECG were:

1. Atrial Fibrillation (AF);
2. First-, second- or third-degree AV block;
3. Left bundle branch block (LBBB);
4. Right bundle branch block (RBBB);
5. Left anterior fascicular block (LAFB).

### Statistical analysis

Continuous variables are reported as means and standard deviations if normally distributed, and as medians and interquartile ranges otherwise. Normal distribution of the variables has been evaluated using Shapiro-Wilk test. Categorical variables are reported as absolute numbers and percentages of the total. To assess statistically significant differences for the comparison of categorical measures, the Chi-square test was used, while for continuous values the unpaired *t*-test was used. A *p*-value < 0.05 was considered to indicate statistical significance. All statistical analyses were performed using R version 3.5.2.

## Results

Baseline characteristics of the final population (498 patients) are shown in Table 1. The average age was 80.4 years, and 252 (50.6%) patients were women. Arterial hypertension was present in 81%, dyslipidemia in 53.5% and diabetes mellitus in 25%. A previous myocardial infarction occurred in 15% of the patients, 16% of the patients had persistent AF, 52% were in NYHA class III or IV and 23.5% presented a history of chronic obstructive pulmonary disease. The average ejection fraction was 59.2% and the mean and maximal aortic gradients were 45.82 ± 14 mmHg and 74.55 ± 21.4 mmHg, respectively. The risk profile was evaluated using Logistic Euroscore II and STS score that were 4.9 ± 4.1% and 4.7 ± 3.8, respectively. Regarding medical therapy, 48.7% of the patients were being treated with a beta blocker, 21% with a calcium channel blocker and 10.6% with amiodarone. The access site was femoral in 471 (94.4%) patients, while 20 cases were performed with a transapical approach (4%) and 7 (1.6%) with a transaortic approach.

**TABLE 1:** Baseline characteristics.

Patients number, *n*	498
Edwards SAPIEN 3 Ultra, *n* (%)	146 (29.5%)
Age (years)	80.4 ± 5
Female, *n* (%)	252 (50.6%)
Height (cm)	165 ± 4.8
Weight (Kg)	71.8 ± 15.4
Body mass index (kg/m^2^)	26.3 ± 7.13
Hypertension, *n* (%)	405 (81%)
Diabetes, *n* (%)	125 (25%)
Dyslipidemia, *n* (%)	266 (53.5%)
NYHA class III or IV, *n* (%)	258 (52%)
COPD, *n* (%)	117 (23.5%)
Previous MI, *n* (%)	75 (15%)
Glomerular filtration rate (mL/min/1.73 m^2^)	53.77 ± 19.25
Logistic Euroscore II	4.9 ± 4.1
STS score	4.7 ± 3.8
Atrial fibrillation, *n* (%)	81 (16%)
Right bundle branch block at baseline, *n* (%)	51 (10%)
**Echocardiographic data**	
LV ejection fraction (%)	59.2 ± 11
Transvalvular mean aortic gradient (mmHg)	45.8 ± 14
Transvalvular maximum aortic gradient (mmHg)	74.5 ± 21.4
**Previous medication**	
Beta blockers, *n* (%)	243 (48.7%)
Calcium channel blockers, *n* (%)	105 (21%)
Amiodarone, *n* (%)	53 (10.6%)

Patients receiving a S3 were 352 (70.5%), while 146 (29.5%) received a S3U. Table 2 shows the comparison of characteristics between the two groups. No significant differences were found in baseline characteristics, echocardiography parameters, and procedural data. Compared to S3U, S3 was implanted deeper into the outflow tract (S3 4.89 ± 1.57 mm vs. S3U 4.47 ± 1.36 mm, *p* = 0.001). Higher implantation was intentional in concordance with recent data indicating the benefits of implanting the valve in a higher position (12, 17).

**TABLE 2:** Comparison of baseline characteristics and procedural data between groups.

Patient number, *n*	498		
	SAPIEN 3 *n* = 352	SAPIEN 3 Ultra *n* = 146	*P-value*
Age (years)	80.6 ± 5.73	80.7 ± 5.29	0.864
Female, *n* (%)	171 (48.5%)	81 (55.5%)	0.324
BMI (kg/m^2^)	26.7 ± 5.25	26.3 ± 4.8	0.921
Hypertension, *n* (%)	304 (86%)	101 (69.2%)	0.107
Diabetes, *n* (%)	94 (26%)	31 (21.2%)	0.791
Dyslipidemia, *n* (%)	176 (50%)	74 (51%)	0.887
NYHA class III or IV, *n* (%)	190 (54%)	76 (52%)	0.789
COPD, *n* (%)	91 (25.8%)	26 (17.8%)	0.146
Previous MI, *n* (%)	49 (14%)	23 (16%)	0.546
GFR (mL/min/1.73 m^2^)	54.9 ± 19.85	52.2 ± 19.48	0.246
Logistic Euroscore II	4.8 ± 4.5	4.33 ± 4.7	0.2658
STS score	3.7 ± 3.5	3.2 ± 3.4	0.346
AF, *n* (%)	61 (17%)	20 (14%)	0.360
RBBB at baseline, *n* (%)	32 (9%)	19 (13%)	0.213
PR interval, ms	168 ± 38.9	175 ± 34.6	0.673
QRS duration, ms	101 ± 25.4	102 ± 23.8	0.854
Moderate or severe LVOT calcium *n* (%)	74 (21%)	32 (22%)	0.843
Membranous septum length (mm)	4.1 ± 2.4	4.2 ± 2.4	0.886
Calcification (grade)	2.35 ± 0.9	2.4 ± 0.94	0.577
Annulus area (mm^2^)	461 ± 85.4	454 ± 83	0.401
**Echocardiography data**			
LV ejection fraction (%)	58.4 ± 10.9	60.6 ± 10.3	0.069
Transvalvular mean aortic gradient (mmHg)	45.6 ± 15.2	46.6 ± 13.5	0.501
Transvalvular maximum aortic gradient (mmHg)	74.3 ± 23	74.8 ± 20.3	0.933
**Previous medication**			
Beta blockers, *n* (%)	182 (51%)	61 (42%)	0.148
Calcium channel blockers, *n* (%)	81 (23%)	36 (24.7%)	0.730
Amiodarone, *n* (%)	39 (11%)	14 (9.5%)	0.642
**Procedural data**			
Percutaneous access			
Transfemoral	331 (94%)	140 (95.9%)	0.819
Transapical	16 (4.5%)	4 (2.7%)	0.270
Transaortic	5 (1.4%)	2 (1.4%)	0.923
**Prosthesis size**			
26 mm	207	71	0.166
23 mm	142	75	0.089
20 mm	3	0	0.264
Predilatation, *n* (%)	37 (10.5%)	16 (11%)	0.889
Postdilatation, *n* (%)	33 (9.3%)	12 (8%)	0.696
Postprocedural PR interval, ms	185 ± 39.4	178 ± 35.6	0.784
Postprocedural QRS duration, ms	111 ± 26.3	110 ± 28.6	0.811
Prosthesis implantation depth (mm)	4.89 ± 1.57	4.47 ± 1.36	0.001

### Rate of conduction disorders

**FIGURE 2:**
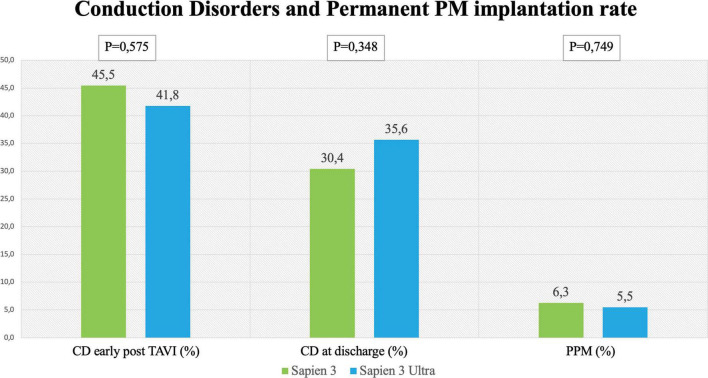
Rates of conduction disorders (CD) Left and central panel shows CD early after the procedure and at discharge with the SAPIEN 3 and SAPIEN 3 Ultra. Right panel shows rate of permanent pacemaker (PPM) implantation after TAVI with the SAPIEN 3 and SAPIEN 3 Ultra.

**FIGURE 3:**
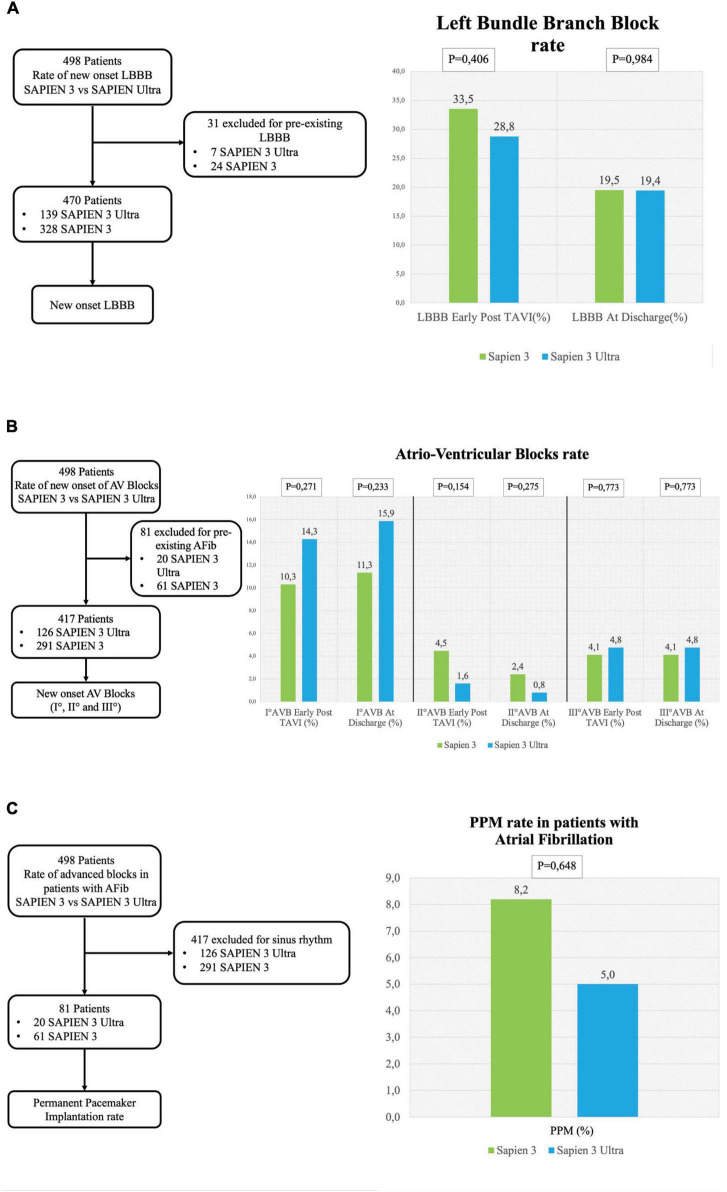
Conduction disorders subanalysis. **(A)** Left flowchart of the analysis for the rate of new-onset LBBB. Patients with pre-existing LBBB were excluded. **(A)** Right left bundle branch block (LBBB) rate early after TAVI and at discharge. **(B)** Left flowchart of the analysis for the rate of new-onset AV blocks. Patients with pre-existing atrial fibrillation were excluded. **(B)** Right rate of I° AV block early after TAVI and at discharge (right graph), II° AV block rate early after TAVI and at discharge (central graph) and III° AV block early after TAVI at discharge (left graph). **(C)** Left flowchart of the analysis for the rate of permanent pacemaker (PPM) implantation in patients with atrial fibrillation. Patients with sinus rhythm were excluded. The graph shows the rate of PPM implantation.

No significant difference in the overall CD rate was found between S3 and S3U patients both immediately after the procedure (S3 45.5% vs. S3U 41.8%, *p* = 0.575) and at discharge (S3 30.4% vs. S3U 35.6%, *p* = 0.348, Figure 2). Figure 3 shows in detail the different types of CD found in the study patients. The rate of new-onset LBBB early after the procedure and at discharge was similar in the two groups (postprocedural LBBB: S3 33.5% vs. S3U 28.8%, *p* = 0.406; LBBB at discharge: S3 19.5% vs. S3U 19.4%, *p* = 0.984) (Figure 3A). Similarly, no difference was found in terms of AV block of different degree between the two groups (Figure 3B).

### Permanent pacemaker rate

The incidence of high-degree AV block requiring PPM implantation was similar between groups (S3 patients 6.3% vs. S3U patients 5.5%, *p* = 0.749) (Figure 2). No significant difference was found in PPM implantation rate in patients with pre-existing AF (S3 8.2% vs. S3U 5%, *p* = 0.648) (Figure 3C). Finally, a subanalysis of AV block occurrence that excluded patients with I° AV block before the procedure did not show any difference among the two groups. Detailed results are shown in Supplementary material.

### Other outcomes including adverse events and paravalvular leakage rate

**FIGURE 4:**
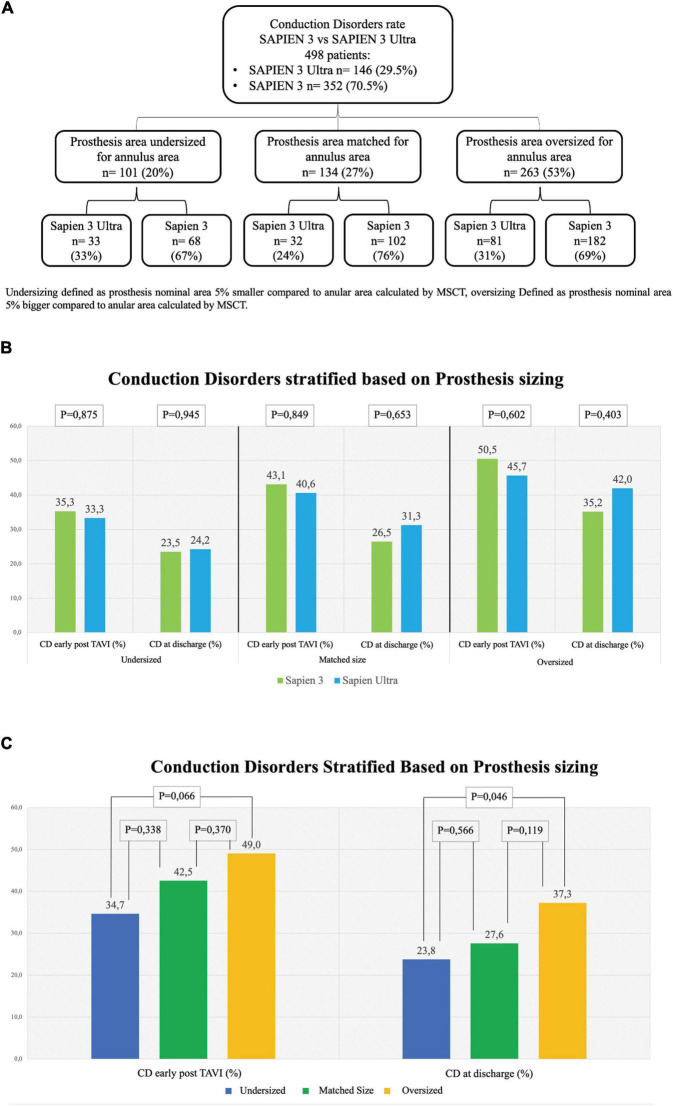
Rate of conduction disorders stratified based on prosthesis size. **(A)** Flowchart of stratification. Undersizing defined as prosthesis nominal area 5% smaller than the annular area calculated by multi-slice computed tomography (MSCT). Oversizing defined as prosthesis nominal area 5% bigger than the annular area calculated by MSCT. **(B)** Conduction disorders (CD) rate early after TAVI in undersized prostheses (left graph), in matched-sized prostheses (central graph) and in oversized prostheses (right graph). **(C)** CD rate based on prosthesis sizing combining together the two generations of valves S3 and S3U early post procedure (left) and at discharge (right). Dark Blue: Undersized group, Dark Green: Normosized group, Yellow: Oversized group.

In our cohort, we found that 101 (20%) patients were treated with an undersized prosthesis, 68 of whom received a S3 (67%) and 33 a S3U (33%). In 134 (27%) patients, the prosthesis was of matched size. Of them, 102 received a S3 (76%) and 32 a S3U (24%). In 263 (53%) patients, an oversized prosthesis was implanted. Of them, 182 were treated with an S3 (31%) and 81 with an S3U (69%). Stratification flowchart is shown in Figure 4A. The comparison between S3 and S3U stratified based on prosthesis size showed no difference (Figure 4B). However, the comparison between oversized und undersized S3 and S3U valves showed a significantly higher rate of CD at discharge in the “oversized” group (37.3%, vs. 23.8% in the “undersized” group, *p* = 0.046) (Figure 4C).

**FIGURE 5:**
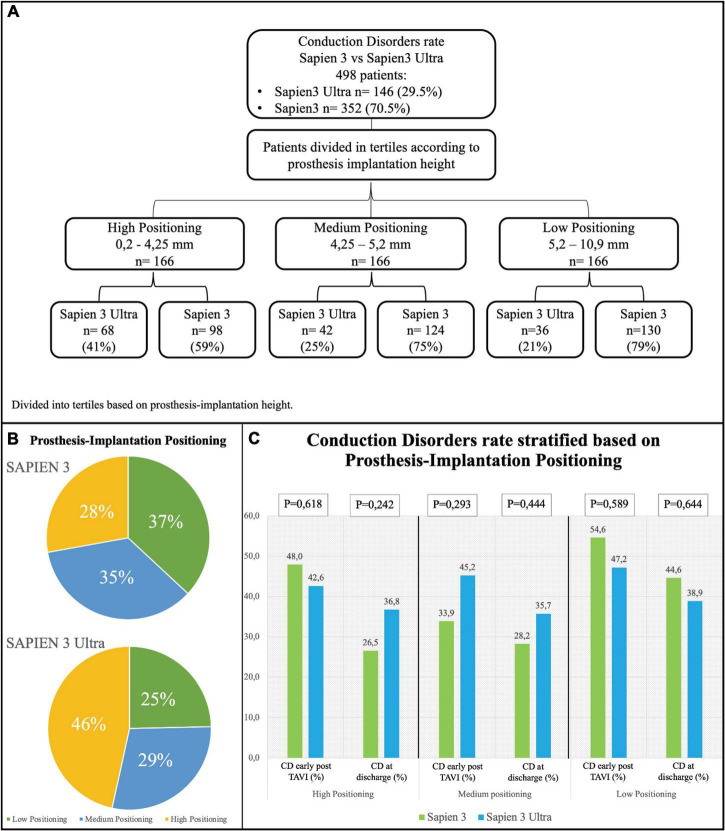
Conduction disorders rate stratified based on prosthesis implantation depth. **(A)** Flowchart of stratification. Prosthesis implantation height was calculated by evaluating angiographic projections during the implantation. The population was then divided into tertiles. **(B)** Distribution of the study population based on valve implantation depth. **(C)** Rate of conduction disorders (CD) early after TAVI and at discharge based on prosthesis implantation depth.

Based on the implantation depth of the THV in the outflow tract, patients were divided in tertiles defining three groups: “high positioning,” “intermediate positioning” and “low positioning” (Figure 5A). A high-positioning was performed in 28% S3 vs. 47% S3U, an intermediate positioning in 37% S3 vs. 25% S3U, and a low positioning in 35% S3 vs. 29% S3U. This indicates that S3U were implanted in a higher position compared to S3 (Figure 5B). A significantly higher CD rate was found with lower implantation position. However, no difference was observed comparing S3 to S3U stratified for prosthesis implantation depth (Figure 5C).

**FIGURE 6:**
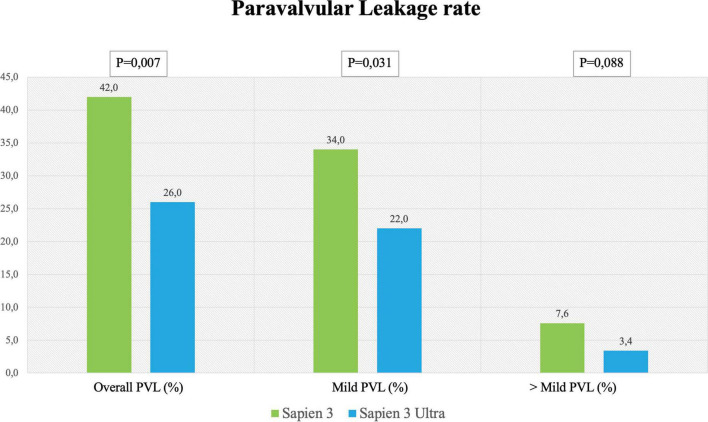
Paravalvular leakage rate. Rate of overall, mild and more than mild paravalvular leakage rate after SAPIEN 3 or SAPIEN 3 Ultra implantation.

In-hospital complications analysis (Table 3) showed low rate of adverse events for both valves with no difference between S3 and S3U except for a significantly lower PVL rate in the S3U patients [S3 148 cases (42%) vs. S3U 38 cases (22%), *p* = 0.007) (Figure 6).

**TABLE 3:** In-hospital complications.

Patients number, *n*	498		
	Sapien 3 *n* = 352	Sapien 3 Ultra *n* = 146	*P-value*
Procedural failure	5 (1.4%)	2 (1.3%)	0.965
In hospital death	2 (0.5%)	2 (1.3%)	0.363
Periprocedural myocardial infarction	4 (1.1%)	1 (0.6%)	0.647
Disabling stroke	2 (0.5%)	1 (0.6%)	0.943
Non-disabling stroke	4 (1.1%)	2 (1.3%)	0.791
Transient ischemic attack	12 (3.4%)	5 (3.4%)	0.993
Major bleeding	6 (1.7%)	3 (2%)	0.791
Major vascular complications	30 (8.5%)	10 (6.8%)	0.548
Paravalvular leakage (overall)	148 (42%)	38 (26%)	0.007
Trivial-mild PVL	121 (34%)	33 (22%)	0.031
> Mild PVL	27 (7.6%)	5 (3.4%)	0.088
Prosthesis thrombosis	4 (1.1%)	0	0.197
In-hospital stay (days)	6.42 ± 3.54	6.17 ± 2.98	0.436

## Discussion

The main results of our study can be summarized as follows:

1. There was no significant difference in terms of CD rate comparing the two latest generations of the balloon-expandable Edwards valve;
2. The rate of PPM implantation was low and comparable between the two groups;
3. CD were relatively frequent after TAVI, and LBBB was the most common CD followed by AV blocks;
4. The S3U valve was implanted in a higher position compared to the S3 valve;
5. The PVL rate was significantly lower with the S3U valve;
6. In-hospital clinical outcome was good and comparable between the two groups.

The primary end point of our study was the incidence of CD after implantation of the latest version of the Edwards balloon-expandable valve (S3U) compared with the previous generation (S3). The prosthesis, which is available in three sizes (20, 23, and 26 mm), features the same bovine pericardium tissue and process as the S3 valve but has a taller, textured polyethylene terephthalate (PET) outer skirt. The main objectives of the new design are the simplification of the procedure due to the new delivery system and a further reduction of PVL risk (7). A recent retrospective study comparing S3 to S3U did not find any difference in terms of 30-day clinical outcomes except for a lower rate of major vascular complications (11.4% vs. 4.5%, *p* = 0,05) and PVL with the S3U (8). However, as the S3U has a “bulkier” and taller PET outer skirt, this could theoretically lead to a higher rate of CD after implantation such as new onset LBBB and high-grade AV block requiring PPM implantation. It is important to highlight that the rate of PPM implantation after TAVI is highly variable in literature and is dependent on many pre-existing anatomical and electrocardiographic factors other than only intraprocedural factors (9). Even if the new design of S3U valve could theoretically look “bulkier” and more risky, the results of our study seem to rule out this possibility showing that the two generation of Edwards balloon expandable valves had a similar CD rate after implantation (Figure 2). The explanation of this similarity could be due the fact that predictors of CD are other than the valve design as previously stated by Sammour et al. (9) Similarly, the need for PPM was low in both groups without any significant difference (6.3% for S3 and 5.5% for S3U, respectively) and slightly less than that reported in the HOMO-Sapien Registry designed for the approval of the S3U valve (8). The low PPM implantation rate observed in our real-world experience matches that of the PARTNER 3 trial designed to evaluate the procedural outcomes in low-risk patients (18).

It is noteworthy that the two patient groups were homogeneous and comparable in terms of baseline and echocardiographic characteristics, excluding selection bias that could affect the results. Moreover, no significant difference was found between groups regarding the grade of valve calcification that is one of the major predictors for new-onset postprocedural CD (19, 20).

Remarkably, if we consider THV sizing, no difference was found in the subanalysis of each of the three groups, “undersized,” “matched” and “oversized,” between the two valves even in presence of a statistically significant difference in the overall rate of CD in the “oversized” group compared to the “undersized” group both early post TAVI and at discharge (Figure 4). These results confirm what has been already reported in literature, i.e., valve oversizing is associated with higher CD rate (20).

The most common CD in our patients after TAVI was LBBB (19.5% in S3 and 19.4% in S3U at discharge), a finding similar to what has been already reported in two previous studies and in a large registry that assessed CD after S3 valve implantation and found a LBBB rate around 20% (19, 21). Although LBBB occurring in fragile patients undergoing TAVI has been shown to reduce 1-year death rate (3.3% vs. 13%, *p* = 0.014), other series gave controversial results suggesting that further studies will be needed to confirm this finding (20, 22, 23). Interestingly, LBBB in our patients was more frequently observed early after the procedure and showed a tendency to regress at discharge as already observed in previous studies (13, 21). Conversely, AV blocks showed a trend to increase at discharge as compared to the early postprocedural time.

There are some procedural aspects that may cause acute injury to the conduction system such as the prosthesis depth into the outflow tract with direct mechanical interaction with the conduction system (19, 24). In our cohort, S3U valves were implanted in a higher position compared to the previous THV generation (Figure 5). A paper published recently by Sammour et al. demonstrated that aiming at a higher implantation position could reduce CD (25). A recent single center study evaluated the predictors of persistence of PM dependency at long term (30 days and 1 year after TAVI). They confirmed that pacemaker dependency after TAVI was strongly associated to implantation depth in relation to membranous septum. Conversely, the membranous septum itself and the type of implanted prosthesis, although previously associated with a higher risk of pacemaker implantation, were not predictive of CD persistence (26).

For the S3U valve, a higher implantation position is favored to the new PET outer skirt that increases the stability of the prosthesis and more importantly provides improved sealing even in a higher implantation position (8).

The reduction of PVL is of importance because several studies and meta-analyses showed decreased survival rates for patients even with mild PVL (13, 18). In the PARTNER trials with the S3, the rate of ≥ mild PVL ranged between 26.3 and 29.5%, while moderate or severe PVL ranged between 0.8 and 3.7% (2, 18). It is noteworthy that our study shows a lower rate of PVL for S3U as compared to S3 (Figure 6). This result is in agreement with the Saia et al. multicenter study that reported a significant PVL reduction with the S3U confirming the advantage of the new sealing skirt of the S3U over that of the S3 (27).

Even if the main objective of this study was to analyze CD occurrence, it should be noted that the clinical outcomes, defined according to VARC-3, were comparable between the two groups indicating the safety of the S3U.

### Limitations

The main limitation of our study is the non-randomized, observational, and monocentric nature of the analysis. Nevertheless, we must state that patients were prospectively and consecutively enrolled in the registry. Second, no statistical adjustment was performed to compare the groups. However, the comparison between the two groups showed very similar profiles with no statistically significant differences in any variable also for what concerns previous drug therapy that could affect the result. For these reasons, no adjustment was deemed necessary. Third, the study population was relatively small. It should be acknowledged that previous reports on CD after S3U implantation focused only on LBBB and PPM implantation rate and did not take in account RBBB and different grades of AV blocks.

## Conclusion

In this retrospective, monocentric series, there was no significant difference in the rate of CD in patients undergoing TAVI with the S3 compared to S3U. Moreover, S3U further reduced the PVL rate without increasing CD or the need of PPM implantation. However, further multicenter, prospective studies including a higher number of patients will be needed to confirm these findings.

## Data availability statement

The raw data supporting the conclusions of this article will be made available by the authors, without undue reservation.

## Ethics statement

The studies involving human participants were reviewed and approved by Centro Cardiologico Monzino. The patients/participants provided their written informed consent to participate in this study.

## Author contributions

GMo ideated the manuscript, analyzed the data, designed the figures, and wrote the draft. PO, GMa, and AM collected the data, analyzed the data, and reviewed the manuscript. FF, LG, and AB performed TAVI procedures and reviewed the manuscript. All authors contributed equally in reviewing the manuscript and contributed to the article and approved the submitted version.
