# Supplementary material for: Conduction disorders after transcatheter aortic valve implantation: A comparison between SAPIEN 3 and SAPIEN 3 Ultra balloon-expandable valves
Source: Front Cardiovasc Med. 2022 Nov 2;9:922696. doi: 10.3389/fcvm.2022.922696 (PMC9666421; doi:10.3389/fcvm.2022.922696)
Supplement: Supplementary file 1 [file Data_Sheet_1.docx]

**Supplemental materials**

**Conduction Disorders After Transcatheter Aortic Valve Implantation: A Comparison Between SAPIEN 3 and**

**SAPIEN 3 Ultra Balloon-Expandable Valves**

Giovanni Monizzi MD^a^, Paolo Olivares MD^a^, Giulio Makmur MD^a^, Franco Fabbiocchi MD^a^, Luca Grancini MD^a^, Angelo Mastrangelo MD^a^, Cristina Ferrari MD^a^, Stefano Galli MD^a^, Piero Montorsi MD^a,b^, Antonio L. Bartorelli MD^a,c^.

**Affiliations:**

1. Centro Cardiologico Monzino, IRCCS, Via Carlo Parea 4, 20136, Milan, Italy.
2. Department of Clinical Sciences and Community Health, University of Milan, Milan, Italy
3. Department of Biomedical and Clinical Sciences “Luigi Sacco”, University of Milan, Italy.

**Corresponding author:**

Giovanni Monizzi MD.

Centro Cardiologico Monzino, IRCCS, Via Carlo Parea 4, 20136, Milan, Italy

Tel: +39 3334433070

E-mail: giovannimonizzi@gmail.com

**Table of content:**

Incidence of Intraventricular Blocks:

1. Atrioventricular blocks Subanalisys…..………...p.3
2. Incidence of Right Bundle Branch Block…….....p.4
3. Incidence of Left anterior Fascicle Block…….…p.5

Procedural characteristics…………………………….…p.6

Implantation depth assessment method…………………p.7

AV blocks rate was similar between groups as already mentioned in the “Results” section of the manuscript (Fig.3 Panel B). A subanalysis was made in patients developing AV differentiating patients with pre existing I° AV blocks and excluding patients with pre-existing AV blocks as shown in figure S1 Panel A. No statistical significant difference in AV blocks rate was found between the two valves taking into account pre existing blocks (FigS1 Panel B) or excluding these patients (FigS1 Panel C) early after procedure or at discharge.


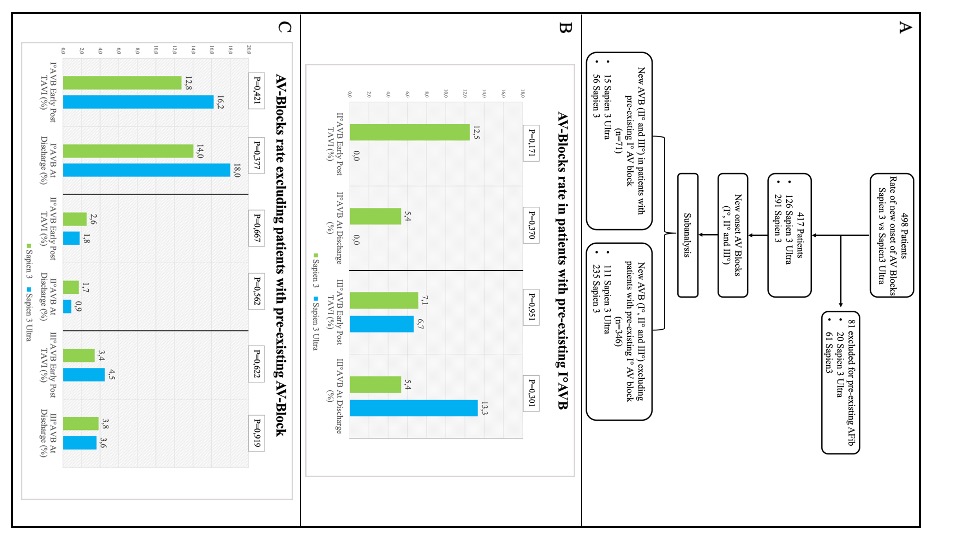


**Figure S1. Panel A:** flowchart of stratification. **Panel B:** AV blocks rate in patients with pre-existing I° AV blocks. **Panel C:** AV blocks rate exluding patients with pre-existing AV blocks.

To evaluate incidence of intraventricular blocks, patients with pre-existing intraventricular blocks were excluded from the sub-analysis (see “Methods” section). No differences were found between the two groups in terms of left bundle branch blocks (LBBB), right bundle branch blocks (RBBB) and left anterior fascicle blocks (LAFB).

Here we highlight how RBBB was less frequent than LBBB without any difference between groups immediately after TAVR (S3 0.6% vs. S3U 1.6%; p=0.338) and at discharge (S3 0.3% vs. S3U 1.6%; p=0.141). (Figure S2)


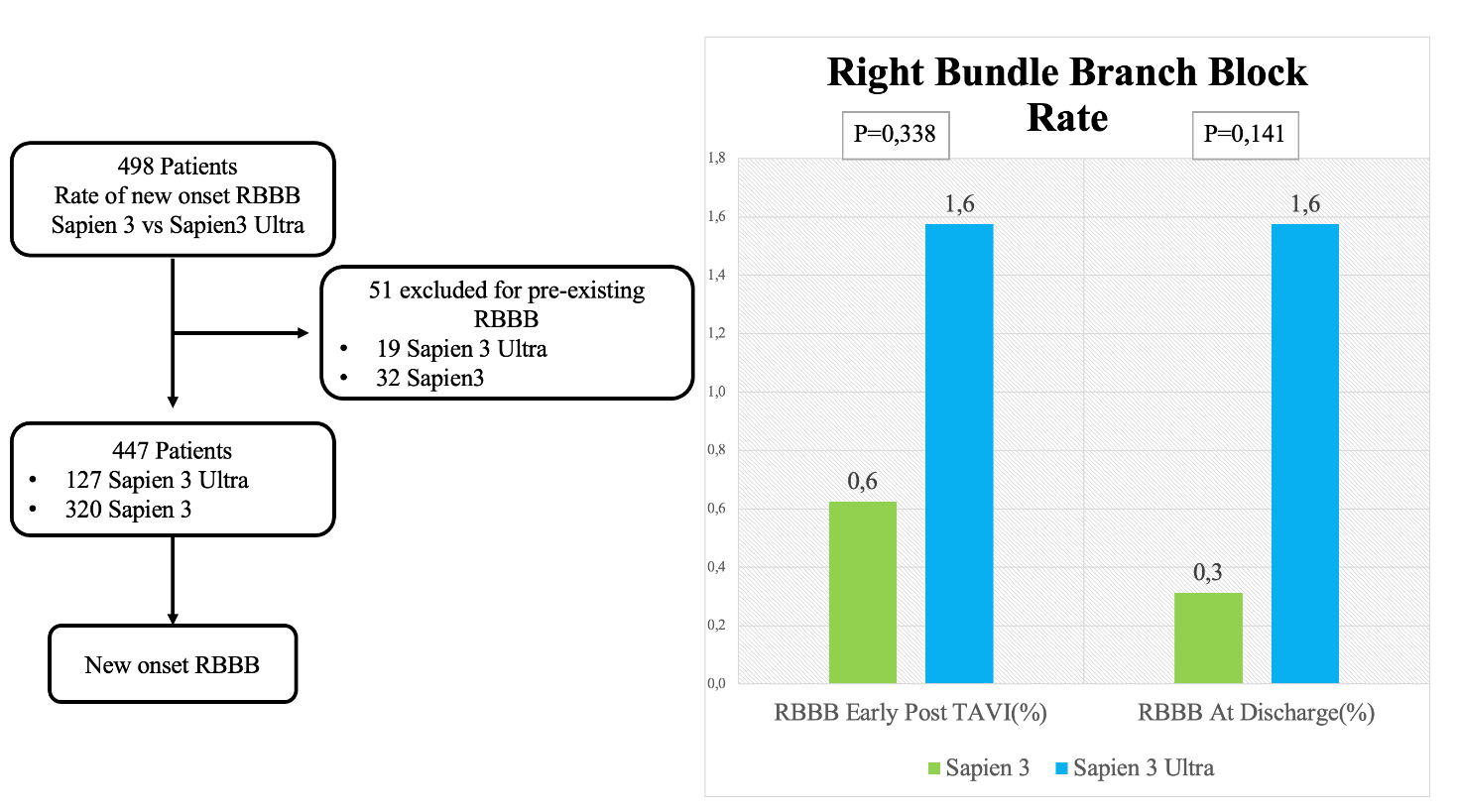


**Fig S2. Left panel:** flowchart of the analysis for the rate of new-onset RBBB. Patients with pre-existing RBBB were excluded. **Right graph:** RBBB rate early after TAVR (**left**) and at discharge (**right**).

No difference was found in terms of LAFB between groups immediately after TAVR (S3 1.9% vs. S3U 2.2%; p=0.843) and at discharge (S3 1.6% vs. S3U 0.7%; p=0.467). (Figure S3)


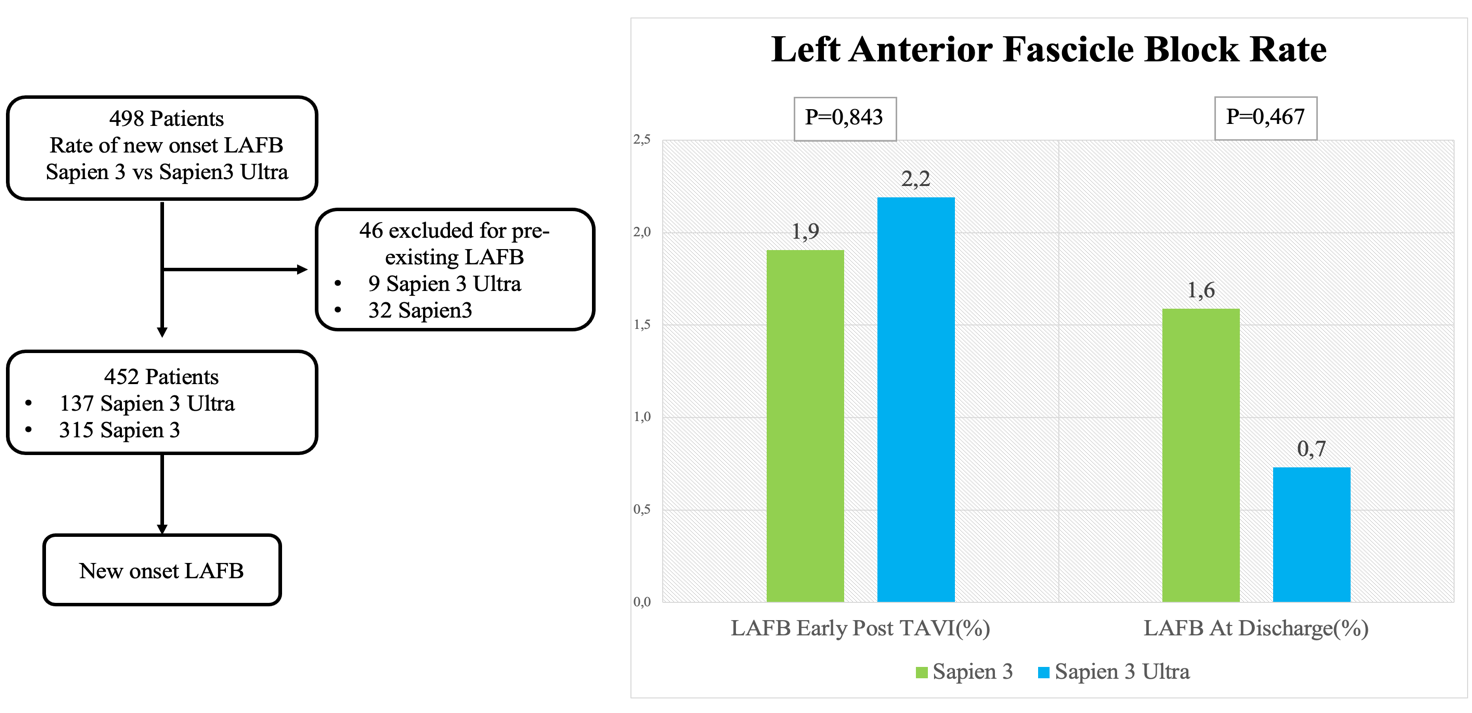


**Fig S3. Left panel:** flowchart of the analysis for the rate of new-onset LAFB. Patients with pre-existing LAFB were excluded. **Right graph:** LAFB rate early after TAVR (**left**) and at discharge (**right**).

| Supplementary Table 1. Comparison of baseline characteristics and procedural data between groups. | | | |
| --- | --- | --- | --- |
| Patient number, n | 498 | | |
| Prosthesis type | SAPIEN 3  n= 352 | SAPIEN 3 Ulltra  n= 146 | p-value |
| **Anesthesia** | | | |
| General Anesthesia | 318 (90.4%) | 129 (88.4%) | 0.831 |
| Deep Sedation | 34 (9.6%) | 17 (11.6%) | 0.528 |
| **Access sites** | | | |
| - Transfemoral | 331 (94%) | 140 (95.9%) | 0.819 |
| - Transapical | 16 (4.5%) | 4 (2.7%) | 0.270 |
| - Transaortic | 5 (1.4%) | 2 (1.4%) | 0.923 |
| Prosthesis implantation depth (mm) | 4.89 ± 1.57 | 4.47 ± 1.36 | 0.001 |

**
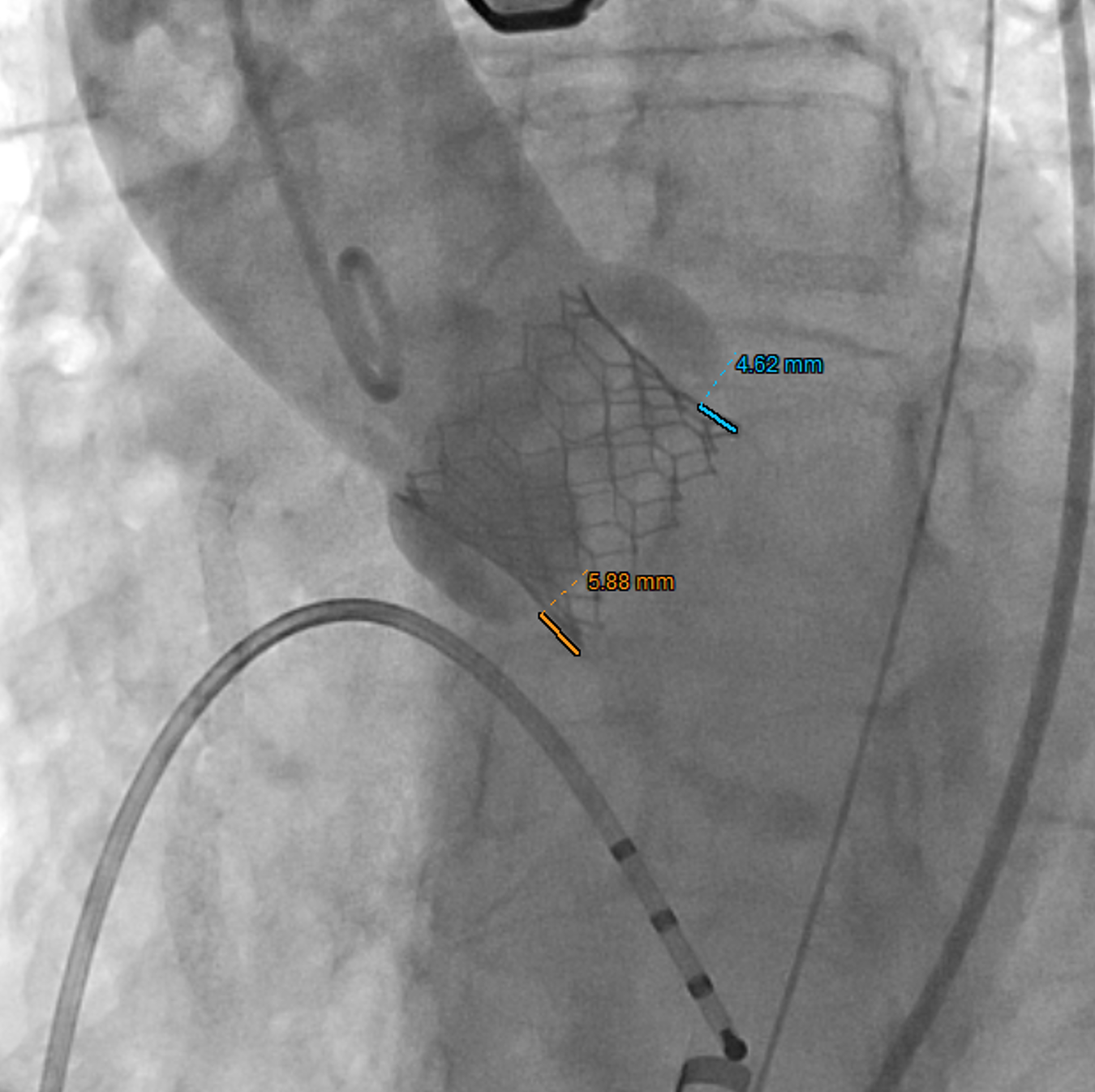
Fig.S4 Implantation depth assessment method**. The THV implantation depth within the left ventricular outflow tract was evaluated by angiographic standard projections during the implantation. The distance between the inferior edge of the cobalt-chromium THV frame and the left and non-coronary cusps was assessed and the mean value of the two measurements was recorded. In the example the measure was 5.25 mm result of the mean of the measure of the non-coronary cusp on the left side (5.88 mm) and of the left coronary cusp (4.62 mm)
